# Chloramphenicol Interferes with 50S Ribosomal Subunit Maturation via Direct and Indirect Mechanisms

**DOI:** 10.3390/biom14101225

**Published:** 2024-09-27

**Authors:** Ting Yu, Fuxing Zeng

**Affiliations:** 1 Department of Systems Biology, School of Life Sciences, Southern University of Science and Technology, No. 1088 Xueyuan Avenue, Shenzhen 518055, China; 2 Institute for Biological Electron Microscopy, Southern University of Science and Technology, No. 1088 Xueyuan Avenue, Shenzhen 518055, China

**Keywords:** chloramphenicol, ribosome biogenesis, 50S precursor, cryo-EM

## Abstract

Chloramphenicol (CAM), a well-known broad-spectrum antibiotic, inhibits peptide bond formation in bacterial ribosomes. It has been reported to affect ribosome assembly mainly through disrupting the balance of ribosomal proteins. The present study investigates the multifaceted effects of CAM on the maturation of the 50S ribosomal subunit in *Escherichia coli* (*E. coli*). Using label-free quantitative mass spectrometry (LFQ-MS), we observed that CAM treatment also leads to the upregulation of assembly factors. Further cryo-electron microscopy (cryo-EM) analysis of the ribosomal precursors characterized the CAM-treatment-accumulated pre-50S intermediates. Heterogeneous reconstruction identified 26 distinct pre-50S intermediates, which were categorized into nine main states based on their structural features. Our structural analysis highlighted that CAM severely impedes the formation of the central protuberance (CP), H89, and H58 during 50S ribosomal subunit maturation. The ELISA assay further demonstrated the direct binding of CAM to the ribosomal precursors, suggesting that the interference with 50S maturation occurs through a combination of direct and indirect mechanisms. These findings provide new insights into the mechanism of the action of CAM and provide a foundation for a better understanding of the assembly landscapes of the ribosome.

## 1. Introduction

There is currently a worldwide threat to infectious disease management and therapy [1,2]. One of the most pressing medical issues today is the ongoing rise in antibiotic-resistant microorganisms [3,4,5]. The urgency of this problem necessitates the identification of new targets to develop additional antimicrobials [6,7,8,9]. The bacterial ribosome, a critical molecular machinery responsible for protein synthesis, is one of the most frequent antibiotic targets [10,11]. In recent years, significant progress has been made in understanding the intricate mechanisms involved in the interaction between ribosome-directed antibiotics and their corresponding targets [10]. These findings provide invaluable information crucial for deciphering their precise mechanisms of action.

Despite the large size of the ribosome, only a limited number of sites are targeted by the current arsenal of antibiotics. On the 30S subunit, antibiotic binding sites are clustered along the path of mRNA and tRNAs. Antibiotics such as edeine [12] and kasugamycin [13] prevent the binding of initiator tRNA to the start codon, thereby hindering the translation initiation [14]. In contrast, most of the antibiotic binding sites on the 50S subunit are present at or near the peptidyl-transferase center (PTC, Figure S1) and inhibit peptide-bond formation by perturbing or preventing the correct positioning of the aminoacylated ends of tRNAs in the PTC. The binding sites of these antibiotics overlap with either the A-site tRNA (e.g., lincosamides [15], oxazolidinones [16], puromycin, and sparsomycin [17]), P-site tRNA (e.g., blasticidin S [18]), or span both the A- and P-sites (e.g., pleuromutilin [19] and streptogramin A [20]). Besides the PTC, certain inhibitors can also interact in the ribosomal exit tunnel adjacent to the PTC [20] or interact with H43/44 of 23S rRNA to disrupt the binding of EF-G, EF-Tu, and IF2 [15]. These studies provide clues for the development of novel therapeutic strategies to combat bacterial infections.

Ribosome biogenesis is another important antibiotic target [11,21,22]. Many antibiotics, including chloramphenicol (CAM), could cause ribosomal assembly defects [23]. CAM is well known as an elongation inhibitor since it inhibits peptide bond formation by preventing the aminoacyl-tRNA 3′ end from binding in the PTC [24,25]. Syroegin et al. recently rationalized that the presence of Ala, Ser, or Thr in the penultimate position of a nascent peptide is required for efficient CAM-induced ribosome stalling [24,26]. In addition, CAM was originally described for the inhibition of ribosome assembly, which can affect the assembling of both small and large subunits [27]. During CAM treatment, the equilibrium between the production of rRNA and ribosomal proteins is dysregulated. Such particles contain precursor forms of rRNA and an incomplete set of ribosomal proteins [28].

Over the past 20 years, studies have examined the effect of 52 different antibiotics on ribosomal subunit formation in six different microorganisms [11]. However, how these antibiotics affect the biogenesis process of ribosomal subunits has been relatively unexplored. In this study, we utilized the advantages of multi-particle cryo-electron microscopy (cryo-EM), combined with the neural network algorithm cryoDRGN, to systematically analyze the 50S ribosomal subunit precursors in *E. coli* under the influence of CAM, obtaining 26 different classes of the ribosomal 50S subunit precursors. By comparing the structures of these precursors, we found that treatment of cells with CAM results in the accumulation of pre-50S precursors, where the formation of the central protuberance (CP) and the final folding of H89 are affected. Direct binding of CAM to the precursors was indicated. Interestingly, CAM also impacts the maturation of H58, a phenomenon rarely observed in previous studies on 50S precursors. Label-free quantitative mass spectrometry (LFQ-MS) analysis showed the upregulation of ribosomal proteins and assembly factors. These results demonstrate the influence of CAM on multiple steps in the assembly of the 50S subunit, greatly facilitating the rational design of new compounds against drug-resistant bacteria.

## 2. Material and Methods

### 2.1. Sucrose Gradient Centrifugation Analysis

*E. coli* MG1655 cells were grown at 25 °C in 200 mL of 2× YT medium until the A_600_ reached 0.2. At this point, CAM was added to a final concentration of 7 μg/mL. Cells without CAM added were used as controls. Cells were then grown for another 2 h at 25 °C and collected by centrifugation at 4000 rpm and 4 °C for 10 min. Cell pellets were resuspended in 1 mL of lysis buffer (30 mM of Tris-HCl, pH 8.0, 60 mM of KCl, 60 mM of NH_4_Cl, and 10 mM of MgCl_2_). DNase I was added to a final concentration of 20 U/mL. Cells were lysed using a homogenizer, followed by centrifugation at 13,000 rpm and 4 °C for 20 min to remove the cell debris. The supernatant was then loaded onto a 20–40% sucrose gradient prepared in lysis buffer and centrifuged at 38,000 rpm in a SW40 rotor at 4 °C for 11.5 h.

### 2.2. Label-Free Quantification Mass Spectrometry Analysis

*E. coli* MG1655 cells were first grown at 25 °C and treated with CAM as described in the sucrose gradient analysis. The cells were harvested, frozen on dry ice, and sent to Beijing Bio-Tech Pack Technology Company (Beijing, China) for label-free quantification mass spectrometry analysis. A total of 1147 proteins were detected, and the fold change of each protein between CAM-treated and untreated samples was calculated by dividing the average normalized area in each sample.

### 2.3. Cryo-EM Sample Preparation and Data Collection

The 35S and 45S peaks were collected separately from the sucrose gradient. Then, the concentration of these two samples was adjusted to 400 nM in lysis buffer, and 2.5 μL of freshly isolated 35S and 45S were applied to glow-discharged quantifoil R1.2/1.3 holey carbon grids with 2–4 nm continuous carbon film on top, respectively. After 30 s of incubation, grids were blotted for 3 s and plunged into liquid ethane using a Vitrobot device (FEI, USA) operated at 4 °C and 100% humidity. Movies were collected on a Titan Krios G3i (FEI, USA), operating at 300 kV, with a Gatan K3 Summit camera (Gatan, Inc., USA). Data acquisition was performed using software EPU 2.3, with a nominal magnification of 81,000×, which yields a final pixel size of 1.05 Å at object scale (defocus ranging from −1.5 μm to −2.5 μm). For each micrograph stack, 30 frames were collected, with a total dose of 30 e^−^/pixel.

### 2.4. Image Processing

Motion correction at the micrograph level was performed with MotionCorr2 [29]. The program CTFFIND 4.1 was used to estimate the contrast transfer function parameters [30]. Image processing, including micrograph screening, particle picking, and 2D and 3D classification and refinement, was performed with RELION 3.1.0 [31]. Non-ribosome particles, 70S and 30S, were all removed by several 2D and 3D classifications. Finally, well-featured particles (233, 637) were combined from three datasets. The refinement of these particles and 3D classification without alignment grouped 91% of the particles into one class, which generated a 3 Å reconstruction for the 50S precursor. Resolutions were reported based upon the gold-standard Fourier shell correlation (FSC) of 0.143 criterion. Figures were prepared in ChimeraX [32].

### 2.5. CryoDRGN Training

Neural network analysis of the pre50S_CAM_ structural heterogeneity was carried out with cryoDRGN [33]. The full stack of 233,637 particles from 3D autorefine in Relion 3.1.0 [31] was directly subjected to one round of high-resolution training in cryoDRGN, with a box size of 256 pixels, 8-dimensional latent variable, and 1024 × 3 architecture for both the encoder and decoder networks.

### 2.6. Occupancy Analysis for the Structural Modules and Classification

For occupancy analysis, 70 volumes were systematically sampled at k-means cluster centers of the latent embeddings. The existing atomic model of ribosome [34] (PDB: 7K00) was used to create masks corresponding to each structural module of the 50S, such as CP, H58, H68, H90–92, and H89. Each of these masks was applied to each of the 70 volumes in turn, and the intensities of all voxels within each masked region were summed. Volumes with similar occupancies in each structural module were merged and finally generated 26 distinct classes.

### 2.7. Identification of CAM Binding in Precursors

To assess the binding of CAM in ribosomal precursors, we used the CAM ELISA kit (lot: XY202406268) purchased from X-Y Biotechnology company. The assay was performed according to the kit manual. Briefly, precursor peaks (25S, 35S, and 45S) were collected from the sucrose gradient and concentrated as described for cryo-EM sample preparation. Aliquots of 25 µL of each sample were mixed with 25 µL of 2× redissolving buffer and incubated with antibody in the dark for 30 min in the ELISA plate. After washing, the substrate solution from the kit was added. The absorbance was measured using a microplate reader (EnSpire) at 450 nm. A standard curve with 0.025, 0.075, 0.255, 0.675, and 2.025 ppb (parts per billion) of CAM was generated to estimate the concentration of CAM in the precursor samples. The mature 70S peak from the same sucrose gradient with the precursors was used as a positive control. The 50S purified from untreated *E.coli* BL21 was used as the negative control. All samples were analyzed in triplicate.

## 3. Results

### 3.1. CAM Modulates Translation and Metabolism-Related Protein Levels in E. coli

**Figure 1:**
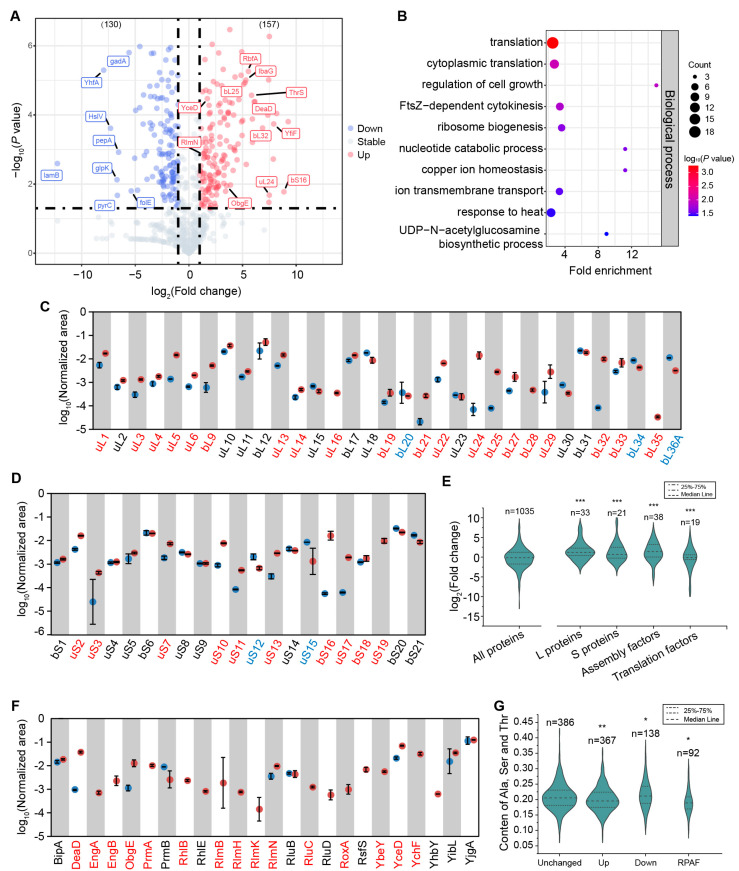
Ribosomal proteins and assembly factors are upregulated in CAM-treated cells. (**A**), Volcano plots showing the fold changes at the protein level. (**B**), The GO pathway analysis of the up-regulated proteins indicates the enrichment of the translation and ribosome biogenesis process. (**C**,**D**), Level of large (**C**) and small (**D**) subunit proteins in CAM^−^ (blue dots) and CAM^+^ (red dots) cells. Proteins without blue dots indicate that these were not detected in CAM^−^ cells. (**E**), Fold changes of ribosomal proteins, assembly factors, and translation factors. L: large subunit. S: small subunit. (**F**), Level of assembly factors in CAM^−^ (blue dots) and CAM^+^ (red dots) cells. (**G**), The total content of Ala, Ser, and Thr in the up- and down-regulated proteins. Unchanged proteins are used as a reference. RPAF: ribosomal proteins and assembly factors. Red texts indicate the up-regulated proteins, blue texts indicate the down-regulated proteins. *, *p* < 0.01; **, *p* < 0.005; *** *p* < 0.001.

CAM has the ability to bind to the PTC and consequently affect translation [25]. To systematically investigate the impact of CAM on the translation process within cells, we first conducted an LFQ-MS analysis of protein levels in *E. coli* MG1655 strains with 7 µg/mL pf CAM for 2h at 25 °C (termed CAM^+^), as previously described [35]. In *E. coli*, ribosome biogenesis tends to be more sensitive at lower temperatures, and the choice of 25 °C helps in capturing potential defects in ribosome assembly that might not be evident at higher temperatures [36]. At the CAM concentration of 7 µg/mL, *E.coli* cells grew moderately slower and exhibited different polysome profiles as compared to untreated cells [27]. Cells without CAM treatment were used as the control (termed CAM^−^, Figure 1). A total of 1146 proteins were identified from the LFQ-MS, of which 676 proteins were detected in both CAM^−^ and CAM^+^ samples. Fifty-one proteins were measured in low amounts across all samples and were discarded from later analysis. Additionally, 389 proteins were only detected in the CAM^+^ samples, while 30 proteins were only found in the CAM^−^ group (Figure S2A).

Differential analysis of the 676 proteins presented in both CAM^+^ and CAM^−^ groups revealed that the levels of 157 proteins were upregulated upon CAM treatment (Figure 1A). Notably, the ribosomal large subunit proteins uL24, bL25, and bL32, small subunit protein bS16, and ribosome assembly factors ObgE, YceD, RlmN, and RbfA showed a more than two-fold increase (Figure 1A). GO enrichment analysis of 370 proteins, including the 157 upregulated proteins and 213 out of 389 proteins exclusively presented in the CAM^+^ group with a *p*-value < 0.05, revealed significant enrichment in cellular pathways associated with ribosome biogenesis and translation (Figure 1B). Furthermore, analysis of all ribosomal protein levels showed that among the 33 detected large subunit proteins, the majority were upregulated in CAM^+^ cells, such as uL5, bL21, uL22, uL24, bL25, and bL32 (Figure 1C,E). Similar results were observed for the 21 small subunit proteins, most of which exhibited upregulation in CAM^+^ cells (Figure 1D,E). Analysis of ribosome assembly factors revealed the same trend as observed with the ribosomal proteins (Figure 1E,F). Conversely, the levels of 130 proteins were found to be downregulated in CAM^+^ cells (Figure 1A) and were predominantly enriched in cellular metabolic pathways (Figure S2B). These results suggest that low concentrations of CAM treatment influence cellular metabolic levels and ribosome status. One possible reason is that under such conditions, cells upregulate ribosome levels to ensure the production of sufficient stress-resistant proteins.

Recently, Syroegin et al. rationalized that the presence of Ala, Ser, or Thr in the penultimate position of the nascent peptide is required for efficient CAM-induced ribosome stalling. If the incoming aa-tRNA carries a Gly residue, CAM-dependent ribosome stalling will be much less pronounced [24]. We thus calculated the percentage of Ala, Ser, and Thr in the upregulated and downregulated proteins. As shown in Figure 1G, compared to the unchanged proteins, the upregulated proteins had lower amounts of Ala, Ser, and Thr, while the downregulated proteins had more of these three amino acids. This is reasonable because the nascent peptides causing CAM-induced ribosome stalling are likely degraded in the cells, meaning proteins with fewer Ala/Ser/Thr residues undergo less degradation. Ribosomal proteins and biogenesis factors also have lower Ala/Ser/Thr residues (Figure 1G), which could further explain the observed upregulation of these proteins.

### 3.2. Treatment with CAM Accumulates Pre-Ribosomal Intermediates

**Figure 2:**
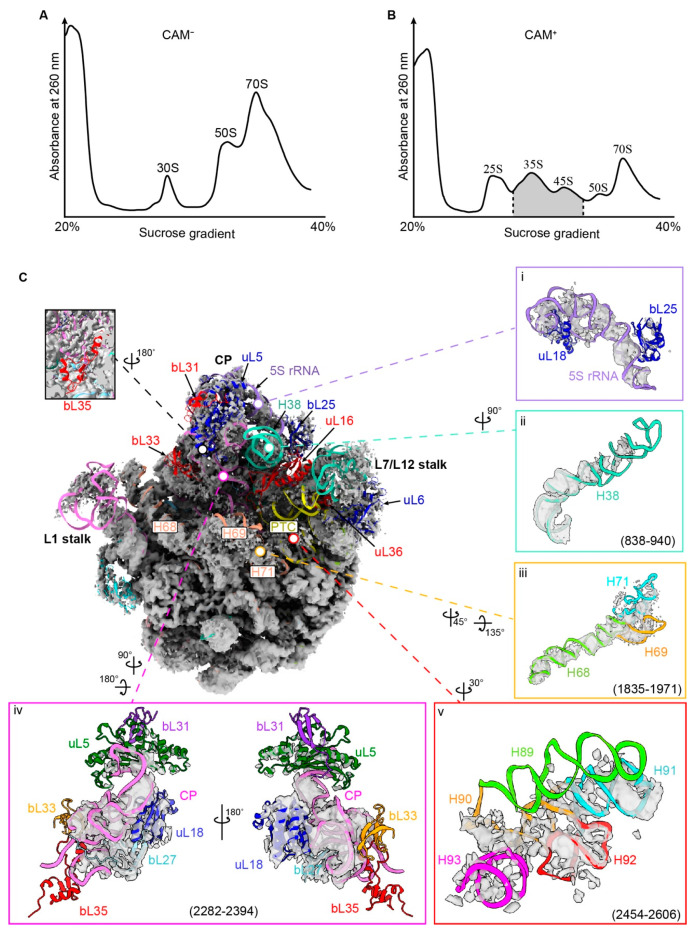
Isolation and structure determination of pre-50S_CAM_. (**A**,**B**) Sucrose gradient analysis of *E. coli* (MG1655) with (**A**) or without (**B**) 7 μg/mL of chloramphenicol. The grey area was collected for cryo-EM analysis. (**C**) Overall structure of pre-50S_CAM_. Panels (**i**–**v**) show the details of each structural module. Coordinates were extracted from 70S (PDB: 7K00) as the references to validate the presence/absence of proteins and rRNA.

Numerous studies reported that CAM affects ribosome biogenesis [11,27,37]. To investigate the impact of CAM on this process, we employed a sucrose gradient to isolate the ribosomal precursors from the *E. coli* MG1655 strain, which was treated with CAM under the same conditions as in the LFQ-MS experiment (Figure 2A,B). Consistent with previous studies [35], precursors of the ribosomal small subunit (25S) as well as those of the large subunit (35S and 45S) were identified (Figure 2B). Peaks were further inspected by cryo-EM or negative staining, which showed good quality for 35S and 45S peaks (Figure S3A,B). Thus, 35S and 45S peaks were collected for cryo-EM data collection (Figure 2B shadow region). However, preliminary 2D classification revealed that the 35S peak contains a higher proportion of 30S particles and smaller pre-50S particles (Figure S3C,D). Therefore, subsequent data collection was primarily focused on the 45S peak. Among the 45S peak particles, approximately 60% of particles represented pre-50S intermediates (Figure S3E). Refinement of the 233,637 particles generated a well-defined pre-50S reconstruction. Further 3D classification without alignment resulted in almost all particles (91%) falling into the same class, which yielded a 3 Å map and was named pre-50S_CAM_ (Figure S3E–G).

As presented in the map, the central region of the 50S exhibited favorable densities, whereas the regions of CP, L1-stalk, L7/L12-stalk, and PTC exhibited much weaker densities (Figure 2C). Specifically, five ribosomal proteins in these regions, including uL16, bL31, bL33, bL35, and uL36, were missing in this structure. Another five proteins, uL5, uL6, uL18, bL25, and bL27, showed weak densities (Figure 2C). For the important rRNA helices, H38 exhibited very high flexibility, resulting in a missing tip near the CP region (Figure 2C(ii)). The 5S rRNA and the H83–87, which constitute the main body of CP, exhibited weak densities (Figure 2C(iv)). In the PTC region, H91–92 had some identifiable densities, but H89, H90, and H93 were too flexible to be observed in the structure (Figure 2C(v)). The long helix H68 showed good occupancy, but the connected H69 and H71 showed very weak densities (Figure 2C(iii)). Collectively, these structural features demonstrated that the 50S precursors obtained in the presence of CAM were primarily in the late stages of maturation.

### 3.3. Identification of the Different Pre-50S Intermediates Impacted by CAM

As indicated above, different regions of the 50S precursor exhibited varying levels of density, reflecting distinct occupancies within these regions. This suggests that pre-50S_CAM_ remains a mixture of different pre-50S intermediates. We then employed cryoDRGN [33], a neural network-based algorithm for heterogeneous cryo-EM reconstruction, to gain insights into the conformational changes occurring in the critical functional domains during the assembly process (Figures S3E and S4A). The pose of each particle was used to train a variational autoencoder (VAE) for heterogeneous reconstruction, with the dimension of the latent variable set to eight (Figure S4A). Volumes were generated by performing k-means clustering on the latent embeddings with k = 70 to ensure that each volume contained approximately 3000 particles (Figure S4B), which yielded the best results for our data. To classify the 70 volumes, densities for key structural features, including the CP region, H68, PTC region (H90–92 and H89), L7/L12-stalk, and H58, were calculated and normalized for further classification (Figure S4C). For the three most important structural modules, CP, PTC (H90–92), and H68, the 70 volumes did not show a good correlation, indicating different effects of CAM on these modules (Figure S4D). In these pre-50S intermediates, most also exhibited additional density on the top of the unfolded CP, PTC, or H68, primarily composed of unfolded rRNA and some assembly factors such as YjgA and ObgE.

Through an occupancy analysis strategy, volumes with high similarity were merged, resulting in the identification of 26 distinct classes from these 70 volumes (Figures S5 and S6). These 26 classes revealed additional details of the assembly intermediates, exhibiting clear structural distinctions. Proteins, including uL5, uL6, uL16, uL18, bL25, bL27, uL30, bL31, bL33, bL35, and bL36, were absent in different classes. Similarly, rRNA helices, including H38, L7/L12-stalk (domain II), H58 (domain III), H67–71 (domain IV), CP and PTC (domain V), and H95/97 (domain VI), exhibited distinct folding statuses (Figures S5 and S6). Based on the folding statuses of these structural features, we categorized the 26 classes into nine main states (Table 1 and Figure S7). Among them, state 1a was the smallest, and its rRNA was defined as ‘core50S_CAM_’ (Figures S1B and S5). When compared to the core region defined by Qin, B. et al. from the in vitro reconstituted pre-50S [38], small differences were observed, including the H33–35a, H38, L7/L12 stalk, H58, H64–66, H75/79, H95/97, and H33–35a (Figure S1B). States 2–4 had CP, H68, and H92 folded, respectively. While states 5, 6, and 8 lacked H92, CP, and H68, respectively. State 7 shared high similarity with state 6, with the exception of additional ObgE. In state 9, most structural modules were assembled (Table 1). These nine states facilitate our subsequent analysis of the influence of CAM on the structure of the pre-50S.

### 3.4. Formation of CP Is Severely Impeded by CAM

Considering that the structures of ribosome precursors obtained to date have predominantly relied on methods involving knockout or enrichment of assembly factors or in vitro assembly, we aimed to mitigate the artificial discrepancies in the enrichment levels of different precursor states caused by these approaches. Thus, we employed pre-50S intermediates obtained from YjgA-ΔNloop cells as references for comparison since the influence of assembly factors on earlier 50S precursors is relatively minimal [39].

During the maturation of 50S, CP assembles just after the core formation, followed by the folding of H68 and PTC [38]. In the present study, states 1, 3, 4, 6, and 7 lacked the density of CP, and two conclusions can be drawn.

**Figure 3:**
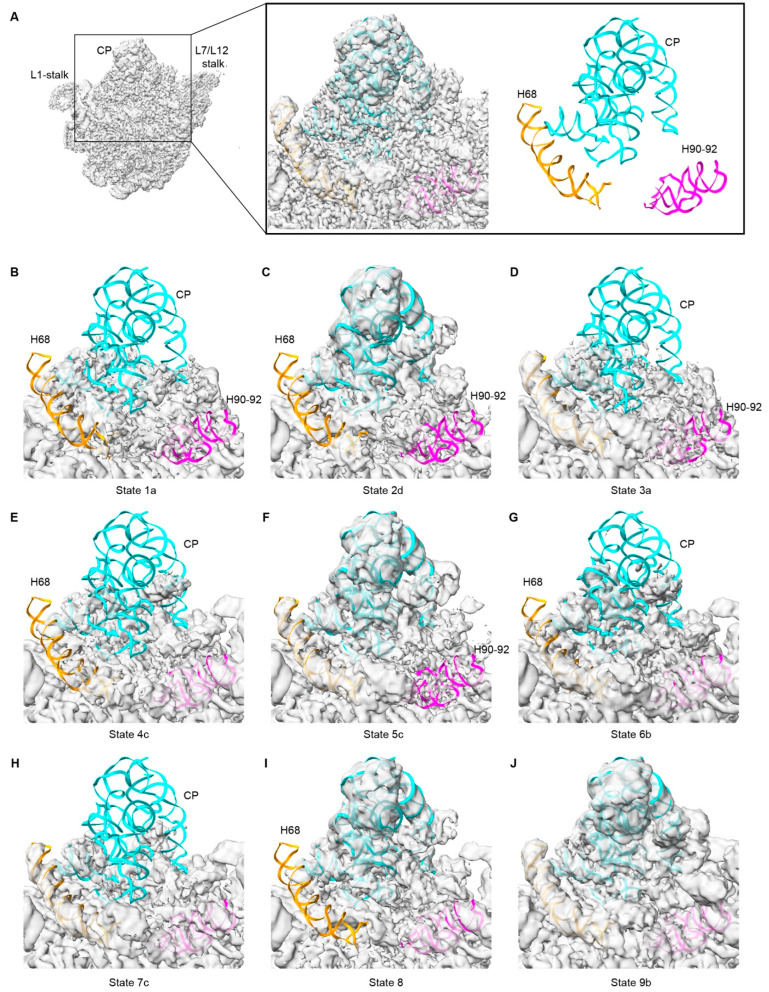
CP is severely impeded by CAM. (**A**) The mature 50S (PDB: 7K00) represents the assembly of CP, H68, and H90–92. (**B**–**J**) Same views as in (**A**) display different conformations of CP, H69, and H90–92 in the nine states.

First, a large number of 50S precursors were accumulated just after the core formation. As described above, states 1a and b only had the core50S_CAM_ assembled (Figure 3 and Figure S5) and accounted for 22% of the particles, which was the highest content among all nine states (Table 1). This state was also observed in YjgA-∆Nloop cells. However, it only comprised less than 4% in the presence of YjgA-∆Nloop [39]. For the in vitro reconstitution, the ‘core’ state had ~15% of particles in the late stages [40]. These results suggested that CAM treatment impedes the transition from state 1 to downstream states. This inhibition could result from CAM directly halting the subsequent folding of state 1 or hindering the folding of downstream states, thereby attenuating the rate of state 1 folding progression.

Second, the folding of H68 and PTC is independent of CP. Among the states lacking folded CP, state 3 (12%) exhibited evident H68 docking (Figure 3D), while state 4 (7%) displayed a prominent H90–92 despite incomplete PTC folding (Figure 3E). States 6 (4%) and 7 (13%) simultaneously exhibited well-defined H68 and H92/93 densities (Figure 3G,H). H68 is believed to dock primarily between CP and the L1-stalk base after CP maturation, while YjgA could bind to the base of CP to undock H68 and facilitate PTC folding [39]. These four states were absent in the 50S precursors from YjgA-∆Nloop cells, indicating that CAM significantly impedes CP folding. This impact may stem partly from CAM-induced alterations in the expression of different assembly factors (Figure 1), while direct CAM binding to rRNA could also partly contribute.

Although CAM primarily binds in the PTC during translation elongation to inhibit peptide bond formation, in the context of the 50S precursor structure, CAM may bind to similar pockets formed by different rRNAs, thus directly impeding the maturation process. While our structure reveals numerous scattered densities near unfolded CP (Figure 3), the exact binding site of CAM remains undetermined. Despite our utilization of cryoDRGN for analyzing the heterogeneous structures of the 50S precursors, its pronounced flexibility remained a primary challenge in structural analysis.

### 3.5. CAM Affects the Final Docking of H89

**Figure 4:**
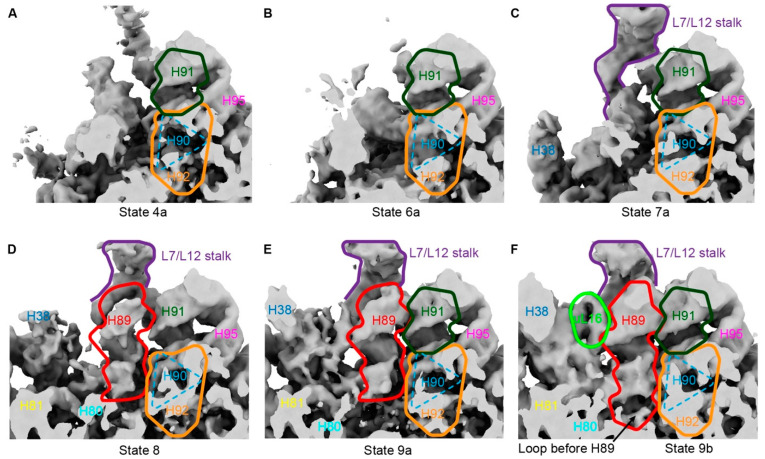
The base of H89 is affected by CAM. (**A**–**F**) Densities around H89 were shown for represented states. H89 is shown in red lines, and the mature base of H89 is labeled with a black arrow in state 9b. Other rRNAs are labeled as structure references.

The incorporation of uL16 and the stabilization of H89 represent late-stage events in PTC formation. After PTC initiation, usually two distinct PTC states can be observed: (i). well-defined density for H90–92 but lacking H89/uL16 density, and (ii). good density for both H90–92 and H89/uL16, which is indicative of complete PTC folding [39]. In YjgA-∆Nloop, when the maturation of H89 is affected, the ratio of these two states is approximately 3:1 [39]. Compared to other studies, such as in ∆YjgA, where the ratio of the two states is 1:1 [39], the state containing immature H89 was significantly increased. Here, when we only considered the states containing H92 (states 4 and 6–9), 73% of the particles lacked H89, and only state 9 had H89 formed. This ratio is similar to the one obtained in YjgA-∆Nloop cells. Therefore, CAM treatment may exert a similar effect, impeding H89 formation even when PTC folding commences (Figure 4). Particularly in state 9a, although folding of H89 was observed, it still lacked proper density for the base (2447–2451/2500–2506) of H89 (Figure 4E,F). Thus, we speculated that CAM may bind to a certain position within the PTC center, creating spatial hindrance and preventing proper placement of H89 at its base.

**Figure 5:**
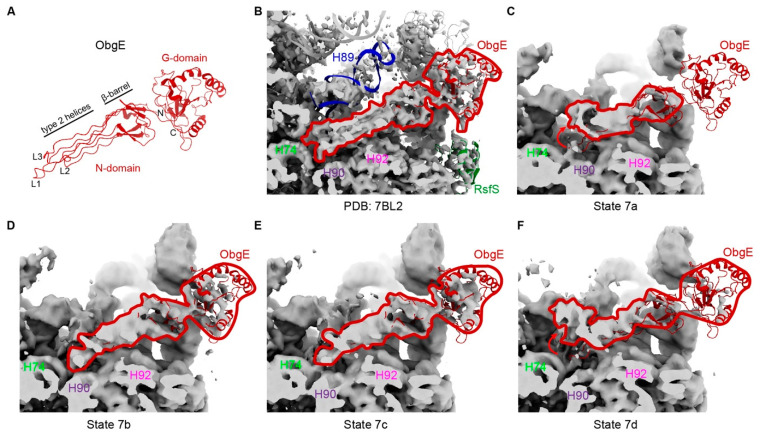
Bent L1–3 of ObgE in CAM–treated 50S precursors. (**A**) Structure characters of ObgE (extracted from PDB: 7BL2). (**B**) Binding of ObgE in 50S precursor under normal conditions (PDB: 7BL2). (**C**,**F**) The tips of ObgE (L1–3) are bent away from PTC. (**D**,**E**) L1–3 point to the PTC as in (**B**).

In state 7a–d, although the CP had not yet formed, the folding of helices (H68 and H92/93) allowed the binding of ObgE to the pre-50S intermediates. As depicted in Figure 5, the bound ObgE exhibited distinct conformations. It has been reported that ObgE coordinates the maturation of the functional core (FC) of the large subunit, including the folding of the rRNA helix H89, along with the incorporation of late assembly proteins bL36 and uL16 [41]. In the reported ObgE complexes, the C-terminal G-domain interacts with the sarcin-ricin loop (SRL), L7/L12 stalk, and RsfS, an anti-association factor that interacts with uL14 and bL19. In some of the structures, RsfS was absent, which was also the case in our states 7a–d, indicating that the interaction between ObgE and RsfS is not essential. However, the involvement of RsfS increased the activity of ObgE in hydrolyzing GTP [41]. Although different conformations were observed in the study of ObgE, interactions with H89 always existed [41]. However, our states 7a–d did not show any density for H89. Moreover, the L1-L3 of ObgE exhibited very high flexibility and showed two distinct conformations: (i). bent away from the PTC, and (ii). points to the PTC (Figure 5). These structural features indicated that L1–L3 plays a crucial role in the maturation of H89. However, as mentioned above, due to the impact of CAM, H89 is more difficult to fold, which allows us to observe different states of ObgE.

### 3.6. Influence of CAM on Other Domains

Besides domain V, domains II-IV and VI also exhibited structure variations, with the exception of domains 0 and I (Figure S6). Domain II encompasses regions that form part of the pre50S core as well as two highly flexible structural modules, H38 and L7/L12-stalk (Figure S1). The folding of H38 occurs synchronously with the formation of the CP, and 5S rRNA serves as an important bridge in the mutual stabilization between the CP and H38 [42]. In our structures, states 1a and 6a lacked H38 (residues 838–940) (Figure S8), while other states reflected the presence of these bases (residues 838–860/917–940) (Figure S8). The impact of CAM on H38 is likely due to the presence of an unfolded CP rather than a direct effect.

Domain III is also the core of 50S precursors forming in the early stage. However, we observed that the H58 in domain III exhibited an unstable structure after treatment with CAM (Table 1). Due to the interaction between H58 and uL2 (Figure S1), poor H58 density was typically observed in 50S precursors only when uL2 was absent, as seen in our state 8. However, in states 1a, 2d, and 6a, only H58 showed significant flexibility, a conformation rarely observed in the 50S precursor structures. A possible reason is that CAM could directly bind to H58, preventing its interaction with uL2 and thereby increasing the structural flexibility of H58.

In our analysis of the 26 classes, only state 8 exhibited suboptimal density in domain IV, characterized by weak density for H64–66 and L2 and a complete lack of density for H67–71 (Figure S5). Additionally, we observed weak density for YjgA at lower thresholds. The poor density of uL2 and H66 may be related to the binding of YjgA, as spatial hindrance exists between the C-terminal domain of YjgA and uL2 proteins. Apart from state 8, all other states achieved full folding of H67, and states 3, 5, 6, 7, and 9 exhibited complete folding of H68–71 (Table 1). These findings suggested that the folding of domain IV is minimally affected by CAM treatment.

### 3.7. Model of CAM’s Action in 50S Maturation

**Figure 6:**
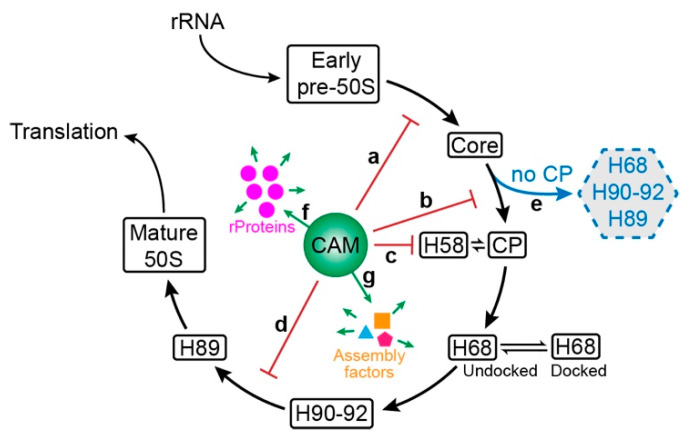
Chloramphenicol affects multiple steps in 50S maturation. Addition of CAM impeded the assembly of early pre-50S (**a**), CP (**b**), H58 (**c**), and H89 (**d**). “Dead-end” ribosomes were also generated (**e**). Ribosomal proteins (**f**) and assembly factors (**g**) were upregulated to overcome the impact of CAM.

Taken together, we suggest that CAM may affect several steps in the 50S assembly process, including CP assembly after core50S_CAM_ formation (Figure 6, step b), H58 folding (Figure 6, step c), and H89 maturation (Figure 6, step d). Although no early pre-50S intermediate was identified, our cryo-EM 2D images for the 35S peak revealed that small particles were enriched (Figure S3A,C), suggesting that CAM may impact the folding of early 50S precursors as well (Figure 6, step a). We also observed that a lot of particles had H68 and H90–92 folded but lacked the CP (Figure 6, step e). These particles might be ‘dead-end’ pre-50S intermediates, which would be degraded by the cells. Due to the mature process being severely affected and the existence of ‘dead-end’ ribosomes, more ribosomal proteins (Figure 6, step f) and assembly factors (Figure 6, step g) were generated to support the survival of cells under the condition of CAM.

## 4. Discussion and Conclusions

Microbial resistance to antibiotics has become a central focus in the development of antimicrobial drugs. The impact of antibiotics on bacteria is multifaceted. Taking CAM as an example, it not only affects the ribosomal translation process but also has a significant influence on bacterial ribosomal biosynthesis [27]. Here, we analyzed a series of ribosomal precursors under low concentrations of CAM and found that CAM could effectively inhibit the folding of CP, H89, and H58 in the 50S subunit.

Two possible effects of ribosome-directed antibiotics on ribosomal subunit formation have been documented: (I). the direct inhibitory effect, where the antibiotic physically obstructs assembly processes, leading to the accumulation of partially assembled ribosomal precursors; (II) the indirect effect, where translation inhibition caused by the antibiotic disrupts the cellular proteome, resulting in secondary accumulation of incomplete ribosomal precursors due to imbalanced availability of ribosomal proteins. The impact of CAM and erythromycin on the assembly of 30S and 50S ribosomal subunits was originally suggested to be primarily due to their indirect effect [43]. This unbalanced synthesis stems mainly from the inhibition of translation elongation by CAM and the regulatory control exerted by ribosomal proteins on their own operons [43]. However, our LFQ-MS results showed that the overall levels of ribosomal proteins are upregulated at a distinct rate (Figure 1). This observation is consistent with the results of two-dimensional electrophoresis [44] and time-course expression of ribosomal proteins [45]. Here, we proposed that the ribosomal proteins may undergo less degradation due to their lower content of Ala, Ser, and Thr, the three amino acids required for efficient CAM-induced ribosome stalling [24,26]. In addition, Koganezawa Y. et al. observed that CAM treatment consistently increases the expression of ribosomal proteins [45]. These two factors may collectively contribute to the upregulation observed in our LFQ-MS analysis.

The non-stoichiometric amount of ribosomal proteins affects 50S ribosomal assembly, but this has only been reported in the context of the absence of specific ribosomal proteins. For instance, the depletion of bL17 leads to the accumulation of several different 50S precursors [46]. Does the increased expression of ribosomal proteins lead to the accumulation of precursors? Our preliminary data showed that overexpression of some ribosomal proteins moderately changed the sucrose profiles. Further experiments are required to investigate the influence of overexpression of ribosomal proteins in the maturation of 50S. The structures of 50S precursors obtained in our study revealed remarkable influences on the CP, PTC, H68, and H58. Together with rRNA helices H82–87, ribosomal proteins uL5, uL18, bL27, and bL31 form a major part of the CP. None of these proteins exhibited decreased expression following CAM treatment. Conversely, uL5 and bL27 were significantly upregulated (Figure 1C). During the maturation process of PTC, the stabilization of H89 is the final step, which requires the assistance of uL16. In our results, a substantial number of particles exhibit difficulties in folding H89, even in the presence of ObgE (states 7a–d) or uL16 (state 9a). The effect of CAM on the folding of H58 is unexpected, as no protein has been reported to be essential for the folding of H58. Although in eukaryotes, the propeller of Erb1 has been reported to bind near H58, it does not influence H58 folding [47]. Based on the observed effects on the CP, H89, and H58, we hypothesize that CAM may also directly bind to the 50S precursor, thereby preventing further folding in these regions. The impaired docking of H68, influenced by the state of PTC, is likely an indirect consequence. The conversion from these precursors to normal ribosomes would require specific protein components absent in CAM-treated particles.

Similar to previously reported findings with erythromycin [48], we also detected the direct binding of chloramphenicol in the precursors (Figure S9). It is important to note that the binding of CAM is not due to contamination from the 50S subunit, as no 50S particles were observed in our cryo-EM results. On mature 50S particles, CAM was reported to bind to PTC, interacting with U2504-U2506, A2061/A2062, and G2505, causing steric hindrance against A-tRNA [49]. A low-affinity CAM binding site was also observed in a hydrophobic crevice at the entrance to the peptide exit tunnel [50]. Based on structural observations, CAM may have additional binding sites on the 50S precursor, such as near the incompletely folded CP or unfolded region around H58. This suggests that, in addition to indirectly affecting ribosome maturation by altering the abundance of ribosomal proteins, CAM can also directly influence precursor folding through its binding to these sites. However, confirmation of the binding sites of CAM in the precursors will require higher-resolution structures and additional biochemical experiments.

In conclusion, our data provide valuable insights into the impact of CAM on ribosomal precursors, highlighting its significant role in modulating the assembly dynamics of ribosomal components. By elucidating the structure of ribosomal precursors disrupted by CAM, we have uncovered mechanisms through which CAM may have direct and indirect effects on ribosome assembly, especially in CP, H89, and H58. This also positions CAM as a useful tool for studying the ribosome assembly process.

## Figures and Tables

**Table 1:** Folding status of the key structural features in all the identified classes.

States	CP	H68	H92	H89	H58	L7/L12-Stalk	Proportion of Particles
1a	−	−	−	−	−	−	3%
1b	−	−	−	−	+++++	−	19%
2a	+++	−	−	−	+++	−	6%
2b	++++	−	−	−	+++	−	3%
2c	−	−	−	−	++	−	3%
2d	++++	−	−	−	+	−	4%
3a	−	+++++	−	−	+++++	−	7%
3b	−	+++	−	−	+++++	−	2%
3c	−	+++++	−	−	+++++	−	3%
4a	−	−	++	−	++++	+	2%
4b	−	−	++	−	++++	−	1%
4c	−	−	++++	−	++++	+++	3%
5a	++++	+++++	−	−	+++++	−	4%
5b	++++	+++++	−	−	+++++	−	4%
5c	++++	+++++	−	−	+++++	−	4%
5d	++++	+++++	−	−	+++++	−	2%
6a	−	++	++++	−	−	−	1%
6b	−	+++	++++	−	++	+	2%
6c	−	++++	++	−	+++	−	1%
7a	−	+++	++++	−	+++++	++++	2%
7b	−	+	++++	−	+++++	++++	2%
7c	−	+++++	++++	−	+++++	++++	4%
7d	−	+++++	++++	−	+++++	++++	5%
8	++++	−	++++	+	−	+	3%
9a	+++++	+++++	+++++	++++	+++	++++	9%
9b	+++++	+++++	+++++	+++++	+++++	++++	1%

−, no density observed. +, with one to four indicating partial folding and five indicating complete folding.

## Data Availability

The cryo-EM map of pre50S_CAM_ has been deposited in the EMDB with accession code EMD-60776. The 26 maps from cryoDRGN and EMD-60776 have also been deposited to Zenodo with the public website: https://doi.org/10.5281/zenodo.11595847 (Published 12 June 2024).

## Supplementary Materials

The following supporting information can be downloaded at https://www.mdpi.com/article/10.3390/biom14101225/s1, Figure S1: Representation of the structure modules in 50S. Figure S2: Down-regulated proteins are enriched in chemotaxis and metabolic processes. Figure S3: Data processing of pre-50SCAM and density evaluations. Figure S4: Classification of the 50S precursors. Figure S5: Overall structure of the twenty-six classes. Figure S6: The rRNA folding in different states. Figure S7: Particle distribution across the nine states. Figure S8: Folding of H38 in CAM-treated 50S precursors. Figure S9: Binding of CAM to 50S precursors.
