# Supplementary material for: Chloramphenicol Interferes with 50S Ribosomal Subunit Maturation via Direct and Indirect Mechanisms
Source: Biomolecules. 2024 Sep 27;14(10):1225. doi: 10.3390/biom14101225 (PMC11505724; doi:10.3390/biom14101225)
Supplement: Supplementary file 1 [file biomolecules-14-01225-s001.zip › biomolecules-3201482-supplementary.pdf]

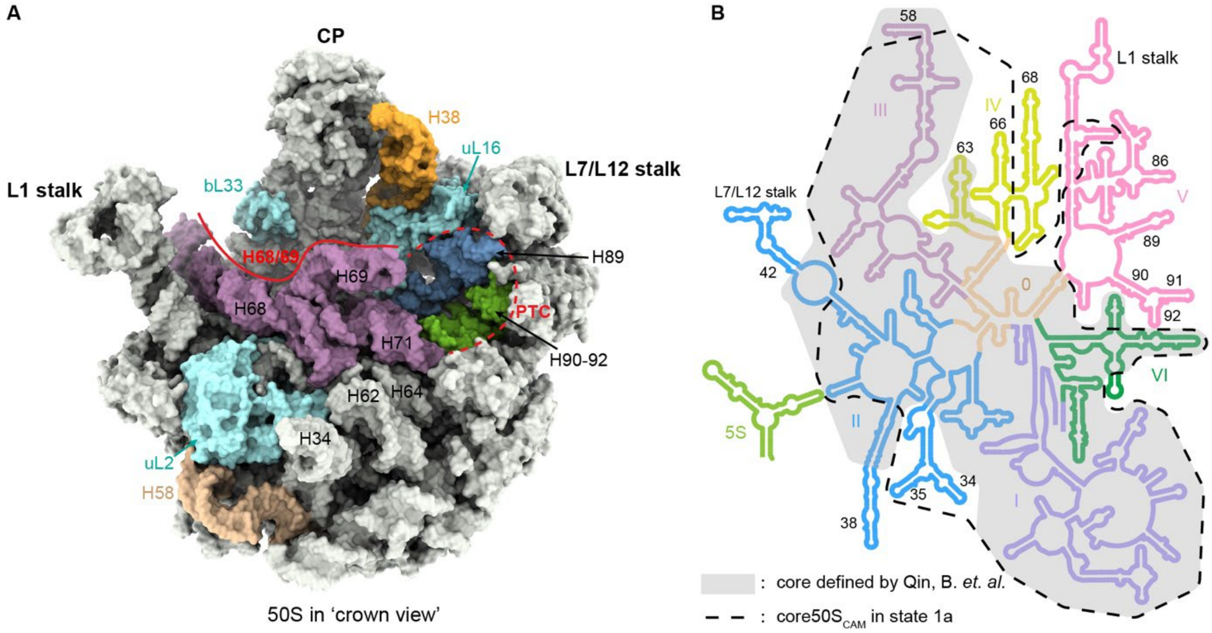

Supplemental Figure S1. Representation of the structure modules in 50S. A, Overall structure of mature 50S (extracted from PDB: 7K00) shown in surface. **B**, The assembly core of 50S precursors defined by Qin, B. et al. and in our state 1a are shown in grey and dashed lines, respectively.

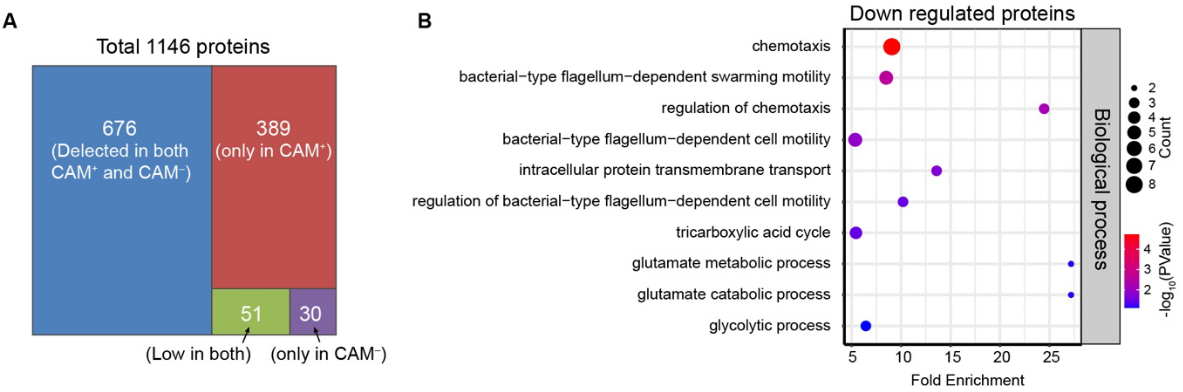

Supplemental Figure S2. Down-regulated proteins are enriched in chemotaxis and metabolic processes. A, Distribution of the 1146 proteins detected in label-free quantitative mass spectrometry. **B**, GO analysis of the down-regulated proteins in CAM$^+$ samples.

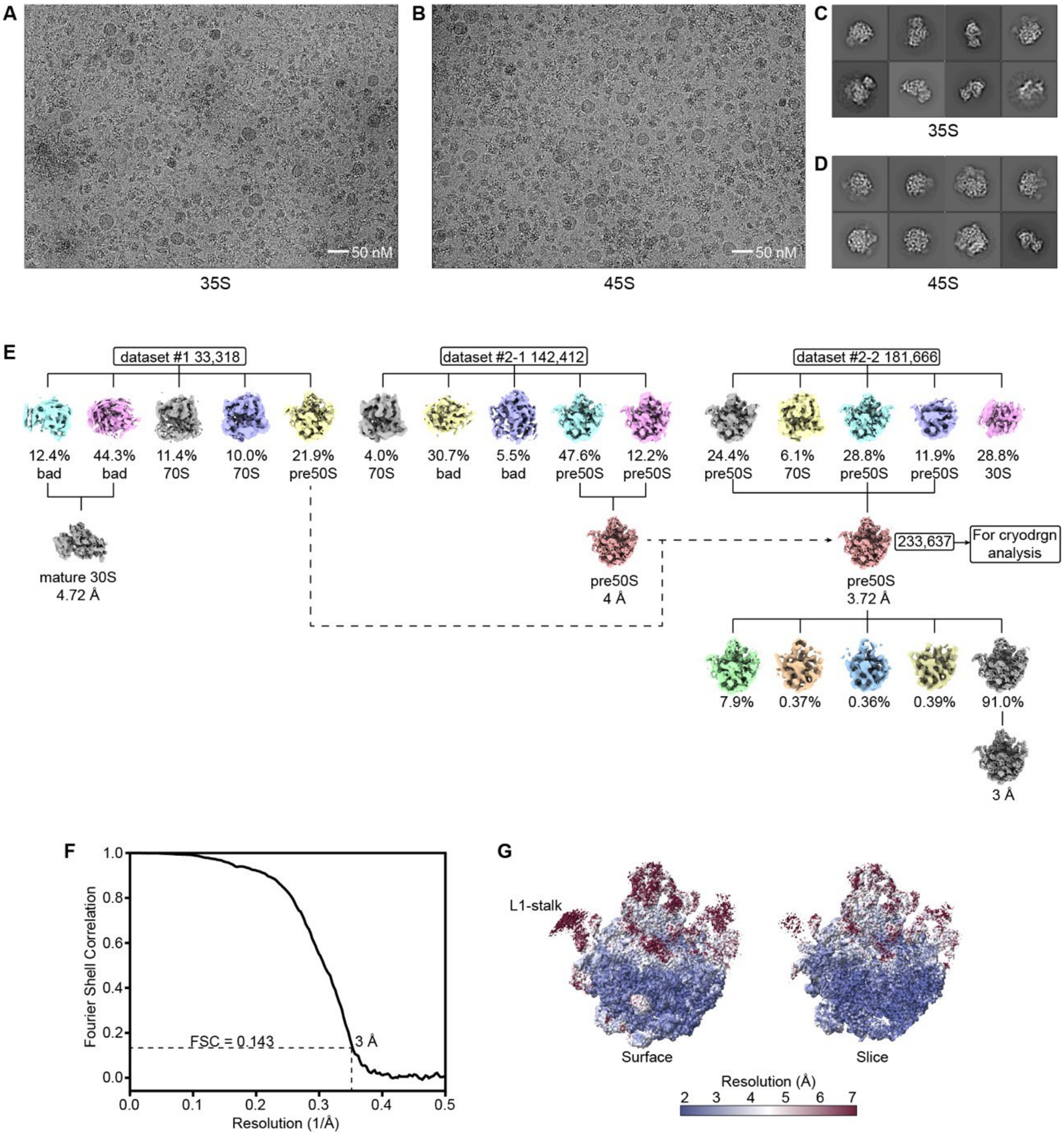

Supplemental Figure S3. Data processing of pre-50S$_{CAM}$ and density evaluations. A and **B**, Representative micrographs for the 35S (**A**) and 45S (**B**) peaks. **C** and **D**, Representative 2D averages for the 35S (**A**) and 45S (**B**) peaks. **E**, Workflow for data processing of pre-50S$_{CAM}$ in Relion. A total of 233,637 particles were selected from three datasets after several rounds of 2D and 3D classifications. A final reconstruction of 3 Å was obtained for the pre-50S$_{CAM}$ intermediates. **F**, Gold-standard FSC curves for the electron microscopy map of pre-50S$_{CAM}$. Resolution is demarcated using the FSC-0.143 criterion. **G**, Local-resolution-filtered map which was colored according to local resolution.

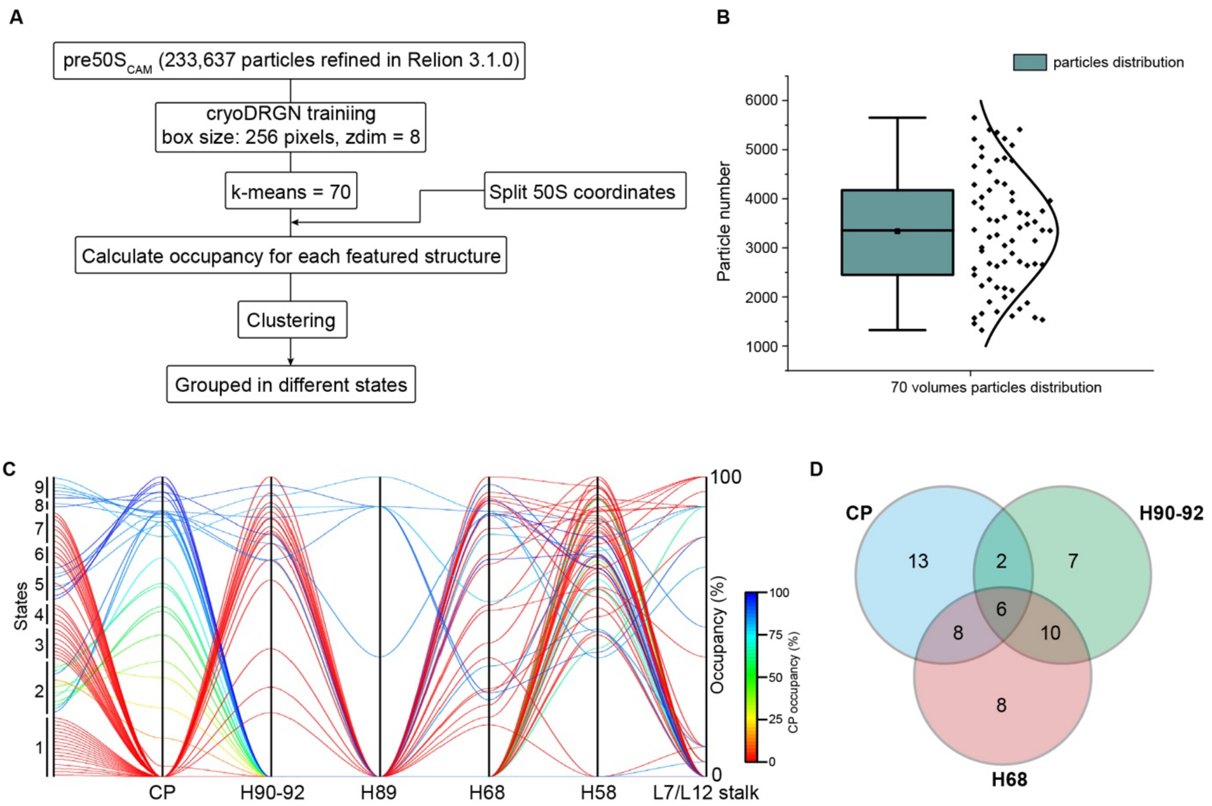

Supplemental Figure S4. Classification of the 50S precursors. A, Strategy of the analysis in cryoDRGN. **B**, Particle numbers in each volume generated by cryoDRNG. **C**, Normalized occupancy of CP, H90-92, H89, H68, H58, and L7/L12 stalk in each volume. Lines were colored in spectrum according to the occupancy of CP. **D**, Veen diagram for the volumes with mature CP, H90-92, and H68, respectively.

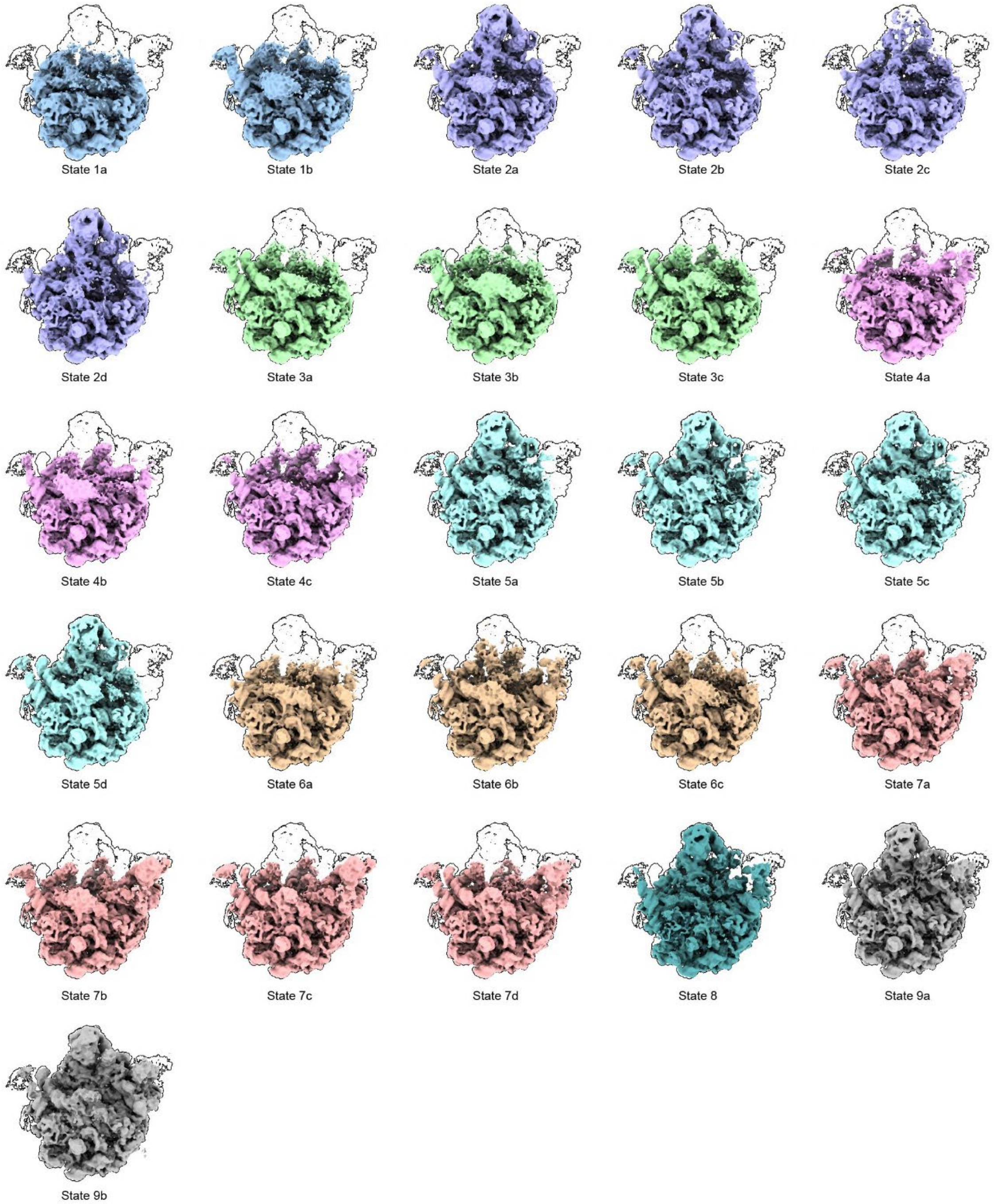

Supplemental Figure S5. Overall structure of the twenty-six classes. The 26 classes obtained from CryoDRGN were grouped into 9 states, with each state represented by a different color. The black outlines represent the contours of the mature 50S subunit extracted from a high-resolution 70S structure (PDB: 7K00).

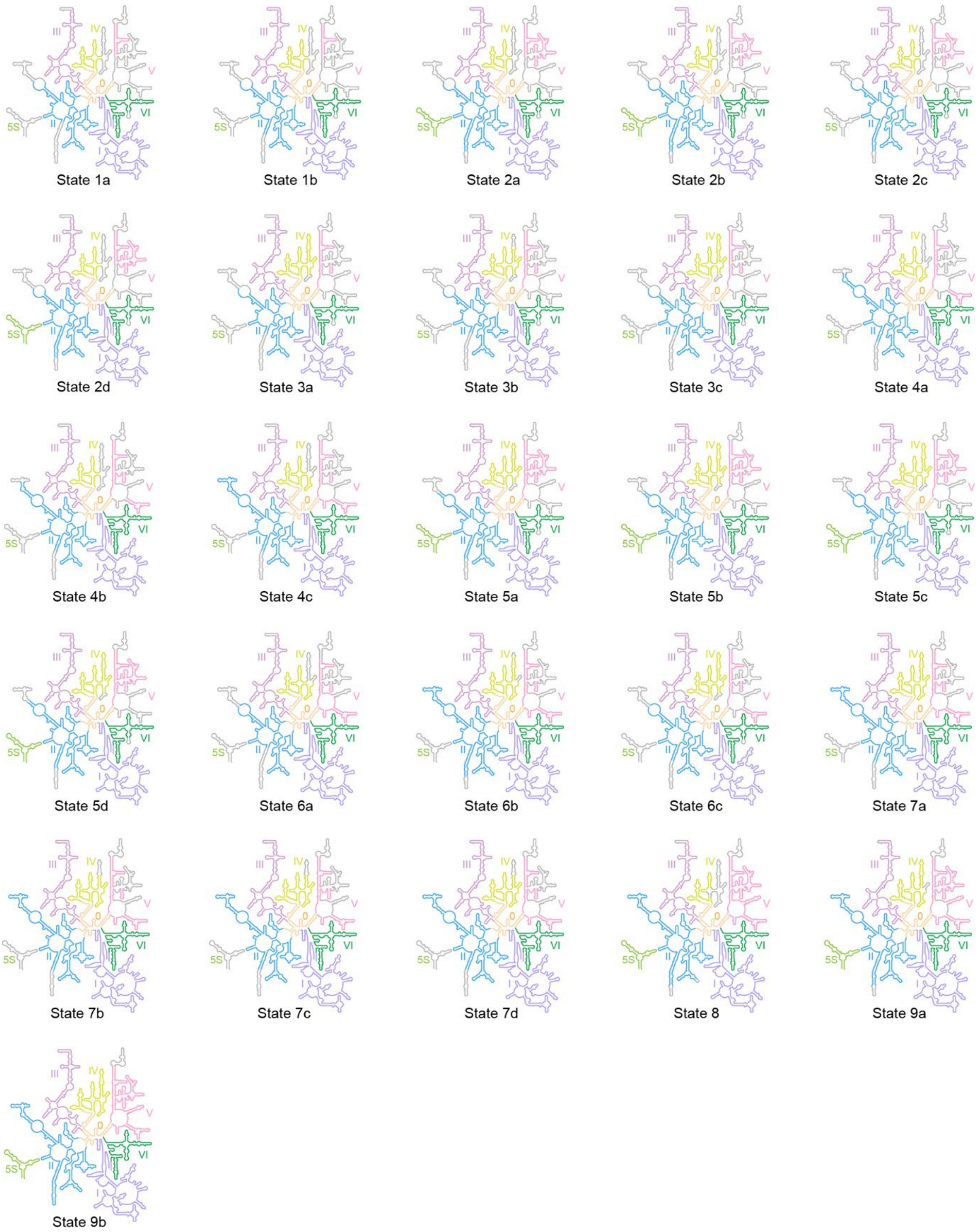

Supplemental Figure S6. The rRNA folding in different states. The 2D structure of the 23S rRNA from the 26 classes obtained by CryoDRGN is represented. Domains 0 to VI are colored in orange, purple, cyan, magenta, yellow, pink, and green, respectively. The 5S rRNA is colored in yellow-green. The folded rRNAs are colored according to their domains, while the unfolded rRNAs are colored in grey.

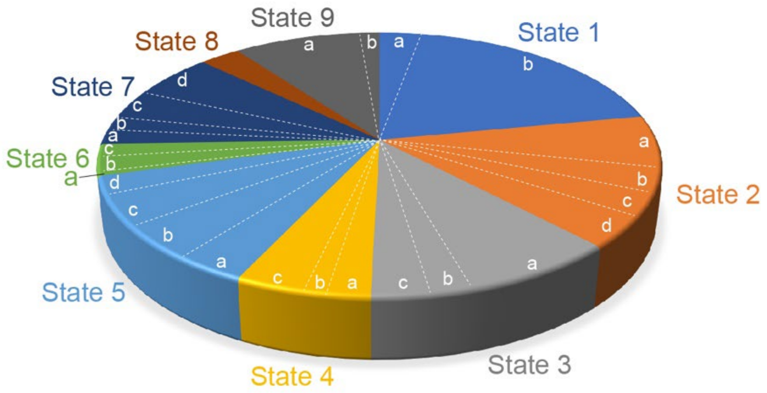

Supplemental Figure S7. Particles distribution in the nine states. The number of particles in each class was counted, and the percentage relative to the total number of particles was calculated and displayed using a pie chart. Each state is represented by a different color.

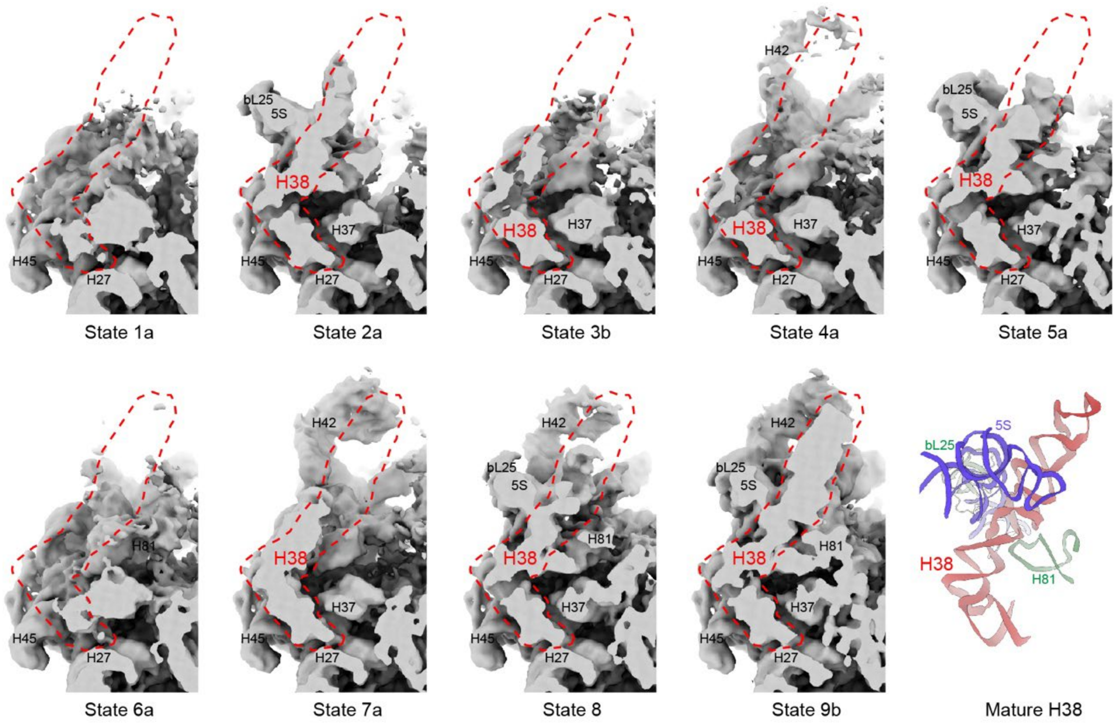

Supplemental Figure S8. Folding of H38 in CAM-treated 50S precursors. A representative map from each state was selected to depict the conformation of H38 in that state. The Cryo-EM maps are shown as a grey surface, with the red dashed line representing the outline of H38 from the mature 50S. Characteristic helices in each state are labeled. H38 from the mature 50S (PDB: 7K00) is displayed as a cartoon, with H38 in red, H81 and bL25 in green, and 5S rRNA in purple.

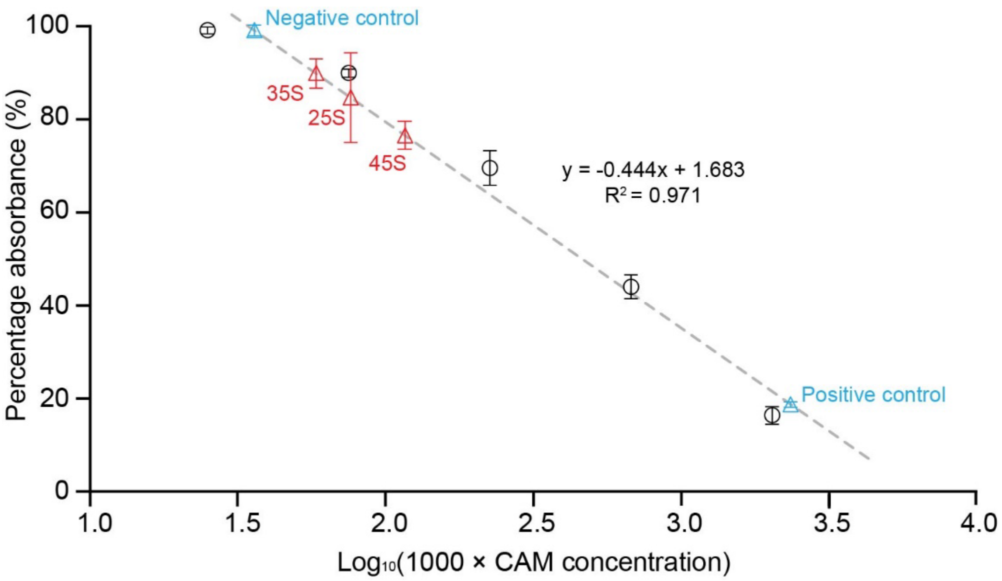

Supplemental Figure S9. Binding of CAM to 50S precursors. The x-axis represents the logarithm of 1000 times the CAM concentration (ppb, parts per billion), and the y-axis shows the percentage absorbance (% absorbance = (mean absorbance of the sample solution / mean absorbance of the 0 ppb standard solution) × 100%). CAM standards (0.025, 0.075, 0.255, 0.675, 2.025 ppb, black circles) were used to generate a standard curve and were linear-fitted (dashed line), with the fitting equation and R^2 value displayed in the figure. The 70S peak was used as a positive control (blue triangle), and 50S subunit purified from *E. coli* grown in the absence of CAM was used as a negative control (blue tringle). Red triangles represent the 25S, 35S, and 45S precursor samples. Each experiment was repeated three times, with error bars indicating the standard deviation.
